# Marginal ice zone dynamics

**DOI:** 10.1098/rsta.2021.0266

**Published:** 2022-10-31

**Authors:** Vernon A. Squire

**Affiliations:** Professor Emeritus, Department of Mathematics and Statistics, University of Otago, PO Box 56, Dunedin, New Zealand

**Keywords:** marginal ice zone, waves, dynamics, morphology

## Abstract

For the best part of my entire career, I have focused on the marginal ice zone, abbreviated to MIZ by most sea ice scientists. Defined perfunctorily by the National Snow & Ice Data Center as the part of the seasonal ice zone where waves, swells and other open ocean processes affect the sea ice, the MIZ habitually extends from the ice edge some 100–200 km into the ice pack with morphology that varies dramatically spatially and with time. In general, the Antarctic MIZ is wider than MIZs in the Arctic, recognizing that increases in the ferocity and incidence of storms and the durability of ice due to global climate change are already affecting the physical attributes of each MIZ. I provide here a somewhat historically tailored preamble to a unique compilation of up-to-the-minute MIZ research in this theme issue that includes the nexus between contemporary theoretical, modelling and experimental projects. A prognosticative synopsis of these projects is also included later in the volume, framed in the context of the ongoing ontogenesis of the research field.

This article is part of the theme issue ‘Theory, modelling and observations of marginal ice zone dynamics: multidisciplinary perspectives and outlooks’.

## 1. Introduction

My personal interest in the marginal ice zone (MIZ) has been to mathematically model and to conduct complementary field experiments on how ocean waves and sea ice interact, drawing in other natural forms of ice such as shore fast ice and vast expanses of continuous, albeit imperfect, i.e. heterogeneous, sea ice. The good news is that much of this work is directly applicable to the MIZ and was done with that in mind, namely to deconstruct MIZ complexity sufficiently that parsimonious mathematical models could be devised to clarify the most significant physics. The bad news is that whereas considerable theoretical progress has been made, there still remains a dearth of field data to test its validity—essentially because *in situ* MIZ experiments are so challenging (and expensive) to carry out, but also that many rudimentary questions are yet to be answered, such as what is the dominant physics responsible for the attenuation of ocean waves propagating within the MIZ?

While studies relating to the physics of the MIZ actually date back to the first half of the twentieth century—with classic articles by notable American geophysicists such as Maurice Ewing, Frank Press, Albert Crary and colleagues—and including prescient theoretical work done at the Courant Institute by the applied mathematician Joseph B. Keller, a more intensive period of activity began in the 1960s, exemplified by publications written by Gordon Robin (Weddell Sea), Ken Hunkins (Arctic Ocean) and David Evans (theory). My principal PhD supervisor, Peter Wadhams, engaged in MIZ research from the 1970s and was one of the scientists who pioneered and steered the multi-year MIZEX programme [1], which took place between 1983 and 1987 in the Bering and Greenland Seas and was one of the largest of the field-oriented research projects originated and supported by the US Office of Naval Research (ONR) Arctic Program. Although I had emigrated to New Zealand by the time the winter MIZEX-87 took place, I was privileged to participate in several of the MIZEX cruises with Peter—in each case focusing predominantly on ocean wave measurements both sides of the ice edge and farther inside the MIZ, with supporting data collected on the nature of the sea ice cover. Our current understanding of the complex dynamics of the MIZ has benefitted and evolved from the excellent science reported in three special editions of the *Journal of Geophysical Research* [2–4] compiled from the MIZEX programme plus a multitude of independent manuscripts published elsewhere.

The comparatively recent *Marginal Ice Zone Program* [5] and the *Arctic Sea State and Boundary Layer Physics Program* [6] in which I participated [7,8] have further added immeasurably and somewhat serendipitously to our knowledge of the MIZ as a result of changes in the Arctic sea ice cover brought about by global climate warming. Both were ONR Departmental Research Initiatives. Like MIZEX, the current theme issue of *Philosophical Transactions of the Royal Society A* is multifaceted, yet it also targets specific features of MIZs that continue to receive enduring attention. Notwithstanding the immense progress made throughout and immediately following MIZEX, especially in understanding MIZ oceanography and meteorology, this emphasis arises because of the staggering complexity of the processes occurring in the MIZ in regard to how it influences and is influenced by the neighbouring open ocean and contiguous interior pack ice. Global climate warming has intensified and increased the frequency of storms since MIZEX as well, recalling that ocean waves and swell are a prime determinant of MIZ morphology—in fact, of whether an MIZ forms at all and of how it matures. Accordingly, three dominant themes emerge in the documented research that follows this preface: wave propagation across the MIZ; floe size distributions; and sea ice dynamics and breakup. A mini review that considers the wider implications of each topic to other areas of science precedes more technically orientated papers, but it is also important to recognize that the substance of the three threads is intertwined with ocean wave impacts having preponderant leverage.

My personal interest in wave-ice interactions was originally stimulated by Gordon Robin and nurtured by Peter Wadhams, although my background initially engendered a greater proclivity towards mathematical modelling as opposed to experiment. This mindset changed rapidly as I discovered the delights of fieldwork in the polar and subpolar seas of both hemispheres and came to believe passionately that the development of theory and the gathering of validating data must be undertaken collaterally. The three reviews [9–11] I have published during my career suggest I am not alone in asserting this opinion, acknowledging that there are innumerable analytical and experimental challenges to comprehending the behaviour of ocean waves interacting with a spatially and temporally variable thermorheological material with a mushy layer structure existing in a variety of natural forms. Needless to say, a parsimonious approach has invariably been taken in the mathematics on the basis that ocean wavelengths are much greater than the sea ice thickness. Methodology is customarily grounded in Greenhill’s insightful thin elastic beam ersatz [12] or refinements therefrom that introduce the Kirchhoff–Love plate, remove the requirement that the ice is thin and has no submergence, exploit dissipative fluid mechanics, incorporate inelasticity, invoke inhomogeneity or transverse isotropy, accommodate imperfections and suchlike.

Although I have published papers that employ more sophisticated rheologies to describe sea ice, e.g. my quixotic foray into thermorheologically simple materials during my PhD and, much later, my work on power law fluids, the austere Kirchhoff–Love plate with or without viscosity has served me and others well. In sum, my obsession has been to solve linear boundary value problems for different topographical situations, e.g. waves impinging on free edges, open and closed cracks, changes of thickness, changes of sea ice freeboard and draught, inhomogeneity, etc. Each of these situations is directly applicable to the MIZ—albeit a rather perfect one, but we have endeavoured to introduce randomness to the distribution of ice floes present in order to increase geophysical credibility. From the outset, my success has been predicated on a steady stream of wonderfully capable PhD students and postdoctoral fellows who have willingly shared their mathematical ingenuity and computational prowess with me. I owe them a great deal but so too does MIZ science, as will be evident in the papers that appear in this volume because our contemporary understanding of ocean wave propagation across the MIZ feeds directly into how the waves reshape it by breaking up floes and are continuously affected by the emerging floe size distribution. This leads to adjustments in the coupling and fluxes between the atmosphere above the MIZ and the ocean beneath, with a consequent transition in its dynamics.

## 2. Conclusion

Taken on balance, it is evident that a disconnect remains between theory and observation despite the considerable advances that have been made in the development of mathematical theory to simulate how ocean wave trains affect and are altered by continuous sea ice sheets, solitary ice floes, random or specified aggregations of ice floes or ice cakes of simple geometry, different manifestations of sea ice embracing contrasting rheologies and so on. Partly this is undoubtedly due to a lack of field data, as such *in situ* experiments are phenomenally difficult to carry out because nature is unsympathetic and rarely obliges with the right conditions. I have personally witnessed the sea ice breaking up just before instruments were installed, ice breakup during deployment that involved a hasty retreat across opening cracks on a snowmobile, an ice floe obliterating our instruments on an adjacent floe by rafting over it and more than 200 km of sea ice being annihilated by freak waves while I was enjoying a beer aboard RV Polarstern in the Weddell Sea. Moreover, the technology to conduct such experiments affordably and expeditiously, viz. satellite-tracked buoys with inertial measurement units that can be interrogated remotely, spaceborne interferometric synthetic aperture radar, etc., is relatively new. But the disconnect is perhaps also due to my erstwhile dogmatic yet naive belief that wave-ice interaction can be approached deterministically, i.e. via randomized scattering, dispersion and attenuation arising from the solution of a boundary value problem defined by prescribed solitary floe behaviour, and we need to accept that this approach does not always work for a medium as capricious as the MIZ.

The corpus of research that follows hereinafter is a concrete step towards broadening our collective thinking about the critical physics of the MIZ for inclusion into fully coupled climate models and, conceivably, Earth system models; a current *Holy Grail* of geoscience that becomes more imperative as climate warming reshapes the polar and subpolar oceans and brings global repercussions.

## Data Availability

This preface does not contain any additional data.
